# Supplementary material for: Identification of photoperiod-induced specific miRNAs in the adrenal glands of Sunite sheep (Ovis aries)
Source: Front Vet Sci. 2022 Jul 22;9:888207. doi: 10.3389/fvets.2022.888207 (PMC9354845; doi:10.3389/fvets.2022.888207)
Supplement: Supplementary file 1 [file Data_Sheet_1.ZIP › Supplementary file/miRDeep2 file.zip!/miRDeep2 file/Novel_Structure/LP42A2/Novel_188.pdf]

Provisional ID	:	NC_019475.2_28898
Score total	:	145
Score for star read(s)	:	3.9
Score for read counts	:	137.3
Score for mfe	:	2.2
Score for randfold	:	1.6
Score for cons. seed	:	
Total read count	:	281
Mature read count	:	231
Loop read count	:	0
Star read count	:	50

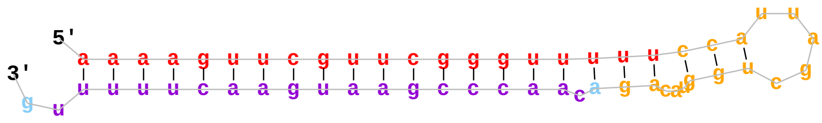

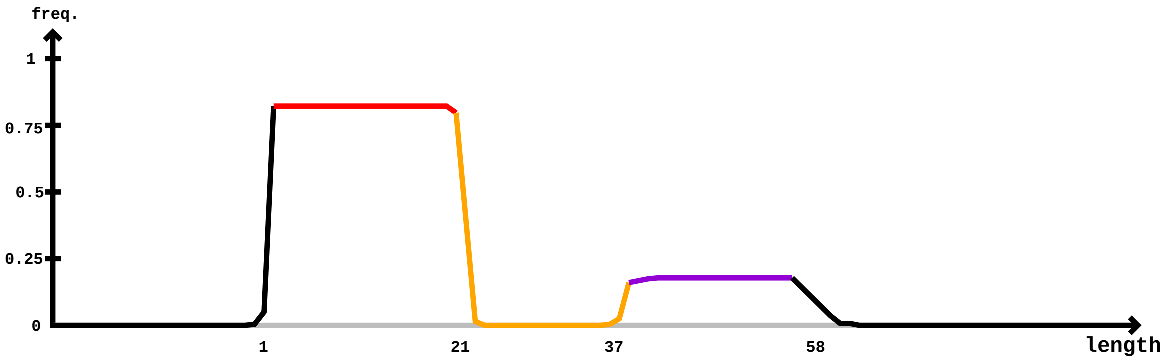

Mature Star

```
5'- agggauguacuggguuggcccaaaaguucguucggguuuuuccauuagcugguacagacaacccgaaugaacuuuuuggccaauucaauauuuccagccucaaggcccagg   -3'    obs
    agggauguacuggguuggcccaaaaguucguucggguuuuuccauuagcugguacagacaacccgaaugaacuuuuuggccaauucaauauuuccagccucaaggcccagg          exp
    ..(((.(((.((((((((((.(((((((((((((((((((((((.....)))...))).))))))))))))))))).)))))))))).))).))).(((....)))..... reads  mm  sample
    ...................cAaaaaguucguucggguuuuu...................................................................... 1      1   seq
    ....................caaaaguucguucggguuuuu...................................................................... 7      0   seq
    ....................caaaaguucguucggguuuuuc..................................................................... 2      0   seq
    ....................Aaaaaguucguucggguuuuuc..................................................................... 4      1   seq
    .....................aaaaguucguucggguuuu....................................................................... 7      0   seq
    .....................aaaaguucguucggguuuuu...................................................................... 130    0   seq
    .....................aaaaguucguucggguuuuC...................................................................... 19     1   seq
    .....................aaaaguucguucggguuuuuc..................................................................... 56     0   seq
    .....................aaaaguucguucUgguuuuuc..................................................................... 1      1   seq
    .....................aaaaguucguucggguuuuucU.................................................................... 4      1   seq
    ........................................................gaAaacccgaaugaacuuuu................................... 1      1   seq
    .........................................................aAaacccgaaugaacuuuuu.................................. 1      1   seq
    .........................................................acaacccgaaugaacuuuuug................................. 1      0   seq
    .........................................................acaacccAaaugaacuuuuug................................. 1      1   seq
    .........................................................aAaacccgaaugaacuuuuugg................................ 2      1   seq
    .........................................................acaacccgaaugaacuuuuugg................................ 1      0   seq
    ..........................................................caacccgaaugaacuuuuu.................................. 2      0   seq
    ..........................................................Aaacccgaaugaacuuuuu.................................. 28     1   seq
    ..........................................................caacccgaaugaacuuuuug................................. 1      0   seq
    ..........................................................caacccgaaugaacuuuuugg................................ 1      0   seq
    ..........................................................Aaacccgaaugaacuuuuuggc............................... 6      1   seq
    ...........................................................aacccgaaugaacuuuuC.................................. 1      1   seq
    ...........................................................aacccgaaugaacuuuuuggU............................... 1      1   seq
    ............................................................acccgaaugaacuuuuuggU............................... 1      1   seq
    ............................................................acccgaaugaacuuuuuggcca............................. 1      0   seq
    .............................................................cccgaaugaacuuuuuggccG............................. 1      1   seq
```
